# Targeting the Wnt/β-catenin pathway and epithelial-mesenchymal transition in gastric cancer: mechanisms, therapeutic strategies, and clinical challenges

**DOI:** 10.3389/fonc.2025.1633699

**Published:** 2025-08-29

**Authors:** Ruixin Shi, Zhenwen Cao, Jie Li, Ru Ji, Zhijuan Guo

**Affiliations:** ^1^ Inner Mongolia Medical University, Hohhot, China; ^2^ Inner Mongolia Medical University Affiliated Cancer Hospital, Hohhot, China

**Keywords:** gastric cancer, Wnt/β-catenin signaling pathway, epithelial-mesenchymal transition, chemoresistance, targeted therapy

## Abstract

Gastric cancer (GC) remains the foremost contributor to global cancer mortality, largely attributable to metastatic dissemination and therapeutic refractoriness. Emerging data implicate the Wnt/β-catenin signaling cascade as a pivotal regulator of epithelial-mesenchymal plasticity, stemness acquisition, and multidrug tolerance in GC. This review delineates the molecular landscape of Wnt/β-catenin aberrations, encompassing genomic perturbations (*NAT10*, *SMC4*), non-coding RNA circuitry (*LINC00665*, *circ0000670*), and (epigenetic reprogramming (e.g., *miR-33b* hypermethylation). Mechanistically, these alterations cooperate with EMT drivers to potentiate metastatic outgrowth and therapeutic evasion. Of particular translational significance are emerging interventions targeting this axis: phytochemicals (Rutin, ginsenoside Rg3) with dual Wnt-CSC inhibitory activity, CRISPR-edited epigenetic modulators (*TET1*/FOXO4), and immune checkpoint blockade-Wnt inhibitor synergism. Notwithstanding preclinical success, clinical implementation faces two critical bottlenecks—pathway pleiotropy and biomarker paucity. To bridge this gap, we propose a precision oncology framework leveraging multi-omics-guided patient stratification, potentially reshaping GC therapeutic paradigms.

## 1 Introduction

The global burden of gastric cancer (GC) poses a substantial challenge to global health. Despite the documented decrease in observed incidence and mortality rates at the global level, it continues to be the third leading cause of cancer-related deaths (1). In Asia, the incidence and mortality rates of gastric cancer continue to be elevated, despite a trend of decline. There is an urgent need for enhanced efforts aimed at achieving early diagnosis and providing accurate treatment (2).

The Wnt/β-catenin signaling pathway has garnered significant attention as a crucial mechanism governing tissue growth, development, and tumorigenesis (3). The Wnt/β-catenin cascade emerges as a pivotal regulator of gastric oncogenesis, with its dysregulation constituting a therapeutic priority. Pathologically sustained activation of this pathway within the GC tumor microenvironment drives invasive and metastatic phenotypes, positioning it among the most consequential molecular drivers of disease progression.

The epithelial-mesenchymal transition (EMT) represents a dynamic cellular reprogramming process enabling epithelial cells to transiently acquire mesenchymal-like properties (4). conferring malignant traits such as: Clonogenic plasticity (tumor cell totipotency); Immune evasion mechanisms; Metabolic adaptability; Therapeutic resistance (5). This phenotypic shift is molecularly characterized by upregulation of mesenchymal markers (e.g., N-cadherin, vimentin) (6).

Critically, Wnt/β-catenin signaling stabilizes cytoplasmic β-catenin, enabling its nuclear translocation to activate oncogenic effectors (*c-Myc*, *cyclin D1*) that coordinate proliferative bursts, stemness maintenance, and EMT initiation (7). Recent advancements in single-cell sequencing have uncovered substantial intratumoral heterogeneity regarding Wnt/β-catenin activation across various gastric cancer subtypes. This variability may elucidate the differing responses to treatment observed in these patients (8). In addition, the interaction between the Wnt signaling pathway and components of the tumor microenvironment—specifically, cancer-associated fibroblasts (CAFs) and tumor-associated macrophages (TAMs)—has been identified as a crucial factor that affects epithelial-mesenchymal transition (EMT) plasticity and drug resistance (9). This review synthesizes mechanistic advances in Wnt/β-catenin-driven GC pathogenesis and critically evaluates emerging therapeutic paradigms.

## 2 Molecular mechanisms of Wnt dysregulation

### 2.1 Genetic drivers

NAT10/USP15(post-translational modification), NAT10 (N-acetyltransferase 10) serves as a crucial member of the GNAT family (10), exhibiting RNA acetyltransferase activity (11). By catalyzing the AC4C modification (12), NAT10 improves the stability of mRNA and enhances its translation efficiency (12–14). NAT10 is implicated in tumor metabolic reprogramming and plays a critical role in the progression, metastasis, and drug resistance of various malignant tumors, including gastric cancer (15–17). Beyond its RNA epitranscriptomic function, NAT10 acetylates β-catenin at specific lysine residues (K49/K312), bolstering its cytoplasmic stability and nuclear translocation, thereby triggering EMT via E-cadherin suppression and N-cadherin/*Snail* induction. Clinically, NAT10 overexpression correlates with reduced overall survival in advanced GC and confers cisplatin resistance (IC50 reduction by 60% upon knockdown) (18);USP15 (Ubiquitin-specific protease 15), a pivotal member of the ubiquitin-specific protease (USP) family (19, 20). The N-terminal region of the protein under consideration contains the DUSP domain in conjunction with two UBL domains. These domains play a crucial role in the stabilization of β-catenin by removing ubiquitin tags, thereby preventing its proteasomal degradation. Furthermore, pharmacological inhibition of this mechanism effectively suppresses hepatic metastasis *in vivo* (21).

SMC4/AGGF1(nuclear transport), *SMC4* (Structural Maintenance of Chromosomes Protein 4) is located at chromosome band 3q25.33 (22). Similarly, it promotes the nuclear import of β-catenin by interacting with importin-α, thereby enhancing the expression of oncogenic targets such as *cyclin D1* and *Bcl-2*. Preclinical studies indicate that knockdown of *SMC4* via RNA interference (RNAi) significantly inhibits tumor growth, resulting in a 70% reduction in xenograft volume (23); *AGGF1*(Angiogenic factor) has unequivocally been shown to enhance the accumulation of β-catenin by phosphorylating GSK-3β at Ser9, thereby inhibiting its kinases’ activity. The blockade of *AGGF1* reverses ascites formation in models of peritoneal dissemination (24, 25).

BZW1 (secretory regulation), *BZW1* (basic leucine zipper protein 1) is classified as a member of the bZIP superfamily (26). As an oncogene, *BZW1* significantly influences the prognosis of patients with gastric cancer by facilitating the migration and invasion of tumor cells. It promotes *Wnt3a* secretion through enhanced ER-Golgi trafficking, thereby amplifying paracrine β-catenin signaling. Importantly, overexpression of *BZW1* is strongly correlated with resistance to 5-fluorouracil (5-FU), exhibiting a 4.2-fold increase in expression levels (9).

### 2.2 Epigenetic & non-coding RNA networks

The dysregulation of noncoding RNAs (ncRNAs) represents a crucial layer of epigenetic regulation over Wnt/β-catenin signaling. Distinct subtypes of ncRNAs exert either oncogenic or tumor-suppressive effects through various mechanisms, including transcriptional, post-transcriptional, and chromatin-modifying processes.

1. LncRNAs play a pivotal role in orchestrating Wnt activation through diverse and multifaceted interactions:Transcriptional regulation: *LINC00665* interacts with YBX1 to enhance the activity of the *Wnt3a* promoter, thereby promoting the nuclear accumulation of β-catenin. Clinically, elevated serum levels of *LINC00665* serve as a diagnostic biomarker for metastatic breast cancer (27). H19 similarly recruits the histone acetyltransferase p300 to β-catenin, thereby enhancing TCF/LEF-dependent transcription. The application of antisense oligonucleotides targeting H19 effectively suppresses lung metastasis in patient-derived xenograft (PDX) models (28); Epigenetic modulation: *TP73-AS1*: Recruits PRC2 to deposit H3K27me3 marks at the *WIF1* promoter, epigenetically silencing this Wnt antagonist in EBV-associated GC. 5-azacytidine treatment restored *WIF1* expression and curtailed lymph node metastasis (29). Critically, H3K27me3-mediated silencing represents histone modification rather than direct DNA methylation; its functional synergy with DNA methyltransferases (DNMTs) in GC requires further validation; miRNA sponging: *LINC01225* sequesters miR-483-3p, thereby derepressing Wnt1. This mechanism has been validated through rescue experiments (30). *ZEB2-AS1* plays a crucial role in stabilizing *ZEB2* mRNA, thereby suppressing E-cadherin expression and activating Wnt5a signaling. Notably, the knockdown of *ZEB2-AS1* leads to a significant reduction of peritoneal metastases by 50% (31).
2. CircRNAs play a significant role in the crosstalk of the Wnt pathway and the remodeling of the microenvironment:circ_0006646 upregulates HMGB1 by sponging miR-665, facilitating β-catenin nuclear translocation. High circ_0006646 expression correlates with shorter progression-free survival (32). Exosomal *circ0000670*, which is induced by cigarette smoke, activates Wnt signaling in precancerous gastric epithelium, thereby promoting dysplasia. This exosome-mediated pathway highlights the role of environmental carcinogens in epigenetic reprogramming (33). However, the receptor-mediated uptake mechanism of exosomal circ0000670 in gastric cells is unknown.
3. miRNAs modulate Wnt activity with precision by directly targeting components of the pathway:Oncogenic miRNAs, specifically miR-20b and miR-324-5p, collaboratively inhibit SUFU, a formidable suppressor of the Wnt signaling cascade. This dual inhibition synergizes with the porcupine inhibitor LGK974, resulting in an 80% reduction in cancer cell viability (34–36); Tumor-suppressive miRNAs: *miR-455-3p* targets ARMC8 to inhibit β-catenin nuclear transport, with mimics demonstrating a suppression of liver metastasis *in vivo* (37, 38). *miR-497* directly interacts in conjunction with the 3’ untranslated region (UTR) of β-catenin. Furthermore, the nanoparticle-mediated delivery of *miR-497* analogs results in a 65% inhibition of tumor growth (30).

### 2.3 Exosome-mediated regulation

Environmental reprogramming:Cigarette smoke promotes the exosomal packaging of *circ0000670*, which subsequently activates the Wnt/β-catenin signaling pathway in gastric precancerous cells (E-cadherin↓, N-cadherin↑). The levels of serum exosomal *circ0000670* are correlated with the progression of dysplasia. GW4869 (exosome inhibitor) reduces hepatic metastases in murine models (33, 39). Critically, GW4869’s non-specific blockade of all exosome secretion may disrupt physiological intercellular communication.

Pathogen-driven dysregulation: Toxins produced by Helicobacter pylori activate Wnt signaling, leading to the induction of epithelial-mesenchymal transition (EMT), marked by the upregulation of *Snail* expression and the concomitant downregulation of E-cadherin levels. This process also promotes cancer stem cell (CSC) features, evidenced by elevated CD44 and Nanog expression. Notably, this phenotype can be reversed through the application of Wnt inhibitors such as XAV939 (40). Furthermore, H. pylori upregulates FRA-1, which collaborates with β-catenin to enhance *c-Myc* transcription while simultaneously recruiting DNMT3A to silence miR-200b, thereby amplifying the crosstalk between Wnt and NF-κB pathways (41).

**Figure 1:**
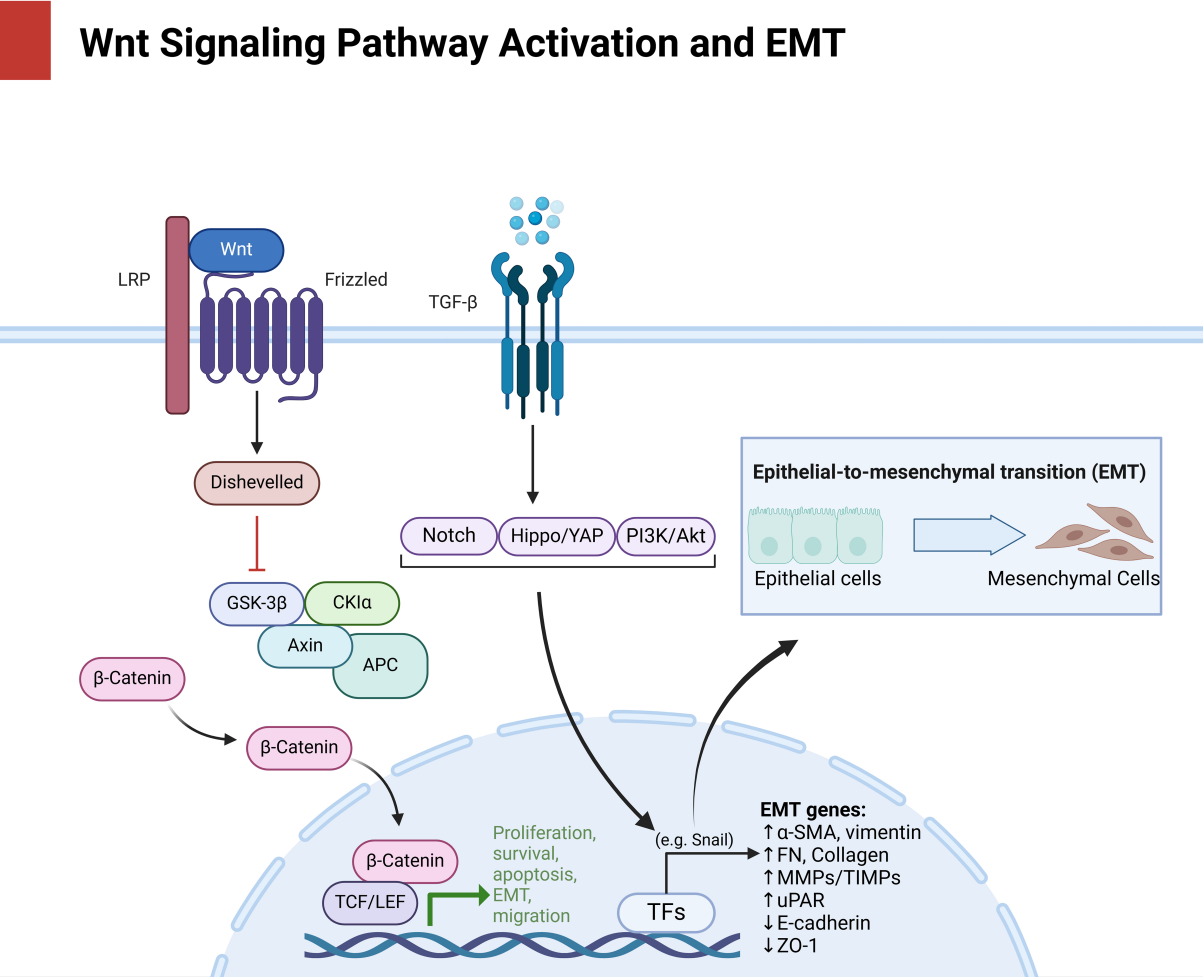
Delineates the multilevel activation of Wnt/β-catenin signaling (LRP/Frizzled/Dishevelled axis) and its convergence with EMT drivers (*Snail*, vimentin, E-cadherin loss), positioning this axis as a nexus for metastatic adaptation and therapy evasion, as mechanistically dissected in subsequent sections.

DNA Methylation and Histone Modification: Promoter Hypermethylation Silences the Tumor-Suppressive Gene Wnt7a, a Defect That Can Be Reversed by 5-Azacytidine (42). *HDAC3*-mediated deacetylation enhances the stability of β-catenin/*TCF4* complexes, while the *HDAC3* inhibitor Honokiol effectively inhibits peritoneal dissemination (43). Concurrently, the suppression of *miR-33b* through promoter methylation and CUL4B-mediated repression exacerbates the activation of Wnt/NF-κB pathways. Furthermore, low levels of *miR-33b* are predictive of resistance to platinum-based therapies (44) **Figure 1**:

## 3 Therapeutic resistance mechanism

Mechanistic studies reveal Wnt/β-catenin signaling as a central orchestrator of therapeutic resistance in GC through two predominant axes:

### 3.1 Cisplatin resistance

*NAT10/TMEM10* Axis: Drives EMT via β-catenin activation, culminating in cisplatin evasion (45). Epigenetic Silencing: *miR-33b* promoter hypermethylation exacerbates multidrug resistance by sustaining β-catenin stability (44). Therapeutic Countermeasure: Ginsenoside Rg3 synergistically suppresses β-catenin, achieving 45% tumor regression in preclinical models (46).

### 3.2 Targeted therapy resistance

*Wnt3a*/*FZD6* Hyperactivation: Induces EMT (E-cadherin↓, vimentin↑) and trastuzumab resistance (47, 48). Rescue Strategy: Wnt inhibitor ICG-001 restores drug sensitivity and triggers apoptosis via TCF/β-catenin complex disruption (48).

Collectively, these findings establish Wnt/β-catenin as a multidimensional resistance hub integrating: Epigenetic reprogramming; Transcriptional rewiring; TME crosstalk. This mechanistic convergence provides a rationale for β-catenin-targeted combinatorial regimens.

## 4 EMT-driven metastasis

### 4.1 Pro-metastatic regulators

Claudin-18-Deficient GC: Upregulates *Snail*2 (core EMT transcription factor) with concurrent Wnt/β-catenin pathway mutations, propelling mesenchymal transition (49). *LINC01225* (Oncogenic lncRNA): Activates Wnt/β-catenin signaling to drive EMT, enhancing proliferative, migratory, and invasive capacities; *LINC01225* silencing ablates EMT phenotypes and suppresses Wnt activity (30). FOXC1-β-catenin Axis: FOXC1 transcriptionally activates β-catenin via promoter binding, triggering EMT (E-cadherin↓, N-cadherin↑, vimentin↑) and metastatic dissemination.β-catenin inhibition reverses FOXC1-driven invasiveness (50). RSPO2 has been found to enhance WNT/β-catenin signaling, thereby promoting the invasion and migration of gastric cancer (GC) cells. Although the dynamic regulatory mechanism of the FOXC1-β-catenin axis in metastatic lesions has not yet been elucidated This finding lends further credence to the hypothesis that components within the Wnt signaling pathway play a crucial role in the regulation of these processes (51).

### 4.2 Metastasis suppressors

The antitumorigenic effects of Mist1 in gastric cancer (GC) have been demonstrated to occur through the modulation of the epithelial-to-mesenchymal transition (EMT) and metastasis. Specifically, Mist1: Inhibits EMT and GC metastasis by suppressing β-catenin transcriptional activity and attenuating Wnt signaling. Overexpression reduces tumor growth and distant metastasis in preclinical models (52). Zic1: Disrupts β-catenin/*TCF4* complex formation, ablating Wnt target gene expression (*c-Myc*, *cyclin D1*) and impairing cell adhesion/invasion.Zic1 activation correlates with improved clinical outcomes The aforementioned effects have been demonstrated to be associated with an improved patient prognosis (53).

### 4.3 Multi-pathway integration

The present study investigates the role of *microRNA-33b*, a tumor suppressor microRNA, in the context of gastric cancer (GC). It is observed that *microRNA-33b* is downregulated in GC, a phenomenon attributed to promoter hypermethylation and the presence of long non-coding RNA (lncRNA)-mediated competing endogenous RNA (ceRNA) networks. It has been shown to inhibit epithelial-mesenchymal transition (EMT) and metastasis by concurrently targeting *NF-κB, MAP8*, and the Notch signaling pathway (44). CUL4B-mediated transcriptional repression and DNA hypermethylation have been shown to work together to suppress *microRNA-33b* (*miR-33b*). This, in turn, has been demonstrated to enhance pro-endometriosis signaling through multiple pathways, including *NF-κB* and *Notch1*. The aforementioned interplay has been demonstrated to promote aggressive tumor behavior (44). In the context of biological processes, *miR-33b* has been identified as a critical regulator that integrates epigenetic, transcriptional, and post-transcriptional signals. This integrated regulation functions to impede a range of processes, including epithelial-mesenchymal transition (EMT) and metastasis. The loss of *miR-33b* initiates a cascade of oncogenic pathway activations.

The following table summarizes the main therapeutic strategies and their mechanisms of action for the Wnt/β - catenin signaling pathway (**Table 1**):

**Table 1:** Therapeutic strategies targeting the Wnt/β-catenin signaling pathway in gastric cancer.

Target type	Specific molecule/pathway	Mechanism of action	Therapeutic strategy
Gene and protein	NAT10	Modification of β-catenin acetylation that enhances its stability and promotes nuclear translocation	NAT10 silencing through RNA interference to attenuate chemoresistance to cisplatin
	USP15	Deubiquitination of β-catenin, maintaining its activity	A small molecule inhibitor, such as PR-619, is capable of blocking the function of USP15
	HDAC3	Deacetylation of β-catenin and the enhancement of its transcriptional activity.	Magnolol inhibits HDAC3, reversing PTD.
Non-coding RNA	LINC00665	miR-203a-3p cloud, Wnt3a/β-catenin signaling activation	Antisense oligonucleotide (ASO) targeting LINC00665
	circ0000670	Exosomal circ0000670 activates the Wnt pathway	Exosome inhibitor (GW4869) or circRNA-specific RNA
	miR-455-3p	Targets ARMC8 and inhibits β-catenin nuclear translocation	miRNA mimics or nanoparticle delivery of miR-455-3p
Natural compounds	Rutin	Delivery of miR-455-3p via miRNA mimics or nanoparticles	Oral chemotherapy regimen (e.g., 5-FU)
	Ginsenoside Rg3	Decreased β-catenin nuclear expression and removal of cisplatin resistance	Intravenous Rg3 nanoparticle injection
	Dihydroartemisinin (DHA)	I.V. Rg3 nanoparticle injection	DHA combined with Wnt inhibitor
Epigenetic regulation	Wnt7a promoter methylation	Hypermethylation inhibits Wnt7a and activates EMT	A demethylating agent (e.g., 5-azacytidine) that restores Wnt7a expression
Signaling pathways	PI3K/AKT-Wnt cross	AKT is phosphorylated by GSK-3β, preventing β-catenin degradation	Dual inhibitor (e.g., LY294002 + XAV-939)
Immune microenvironment	EHD3	Activation of the immunosuppressive TME through the Wnt pathway	EHD3 antibody plus PD-1 inhibitor

## 5 Immune evasion orchestrated by Wnt/EMT axis in gastric cancer

The Wnt/β-catenin-EMT axis drives immunosuppression in gastric cancer through three synergistic mechanisms:

1. T-cell Exclusion: EMT-transformed cells secrete CXCL12 to establish physical barriers that block CD8+ T-cell infiltration, particularly in diffuse-type GC, correlating with “immune desert” phenotypes (54, 55). Concurrently, hsa_circ_0001479 (upregulated in GC) inhibits CD8+ T-cell recruitment via the miR-133a-5p/DEK/c-Myc axis while activating Wnt signaling, forming a feedforward loop to sustain immune evasion (56).
2. Myeloid Reprogramming: Wnt activation induces IL-6/G-CSF secretion, expanding polymorphonuclear myeloid-derived suppressor cells (PMN-MDSCs) that express ARG1/iNOS to suppress T-cell function in peritoneal metastases (54, 57). The orphan GPCR GPR176 (overexpressed in GC) further polarizes macrophages toward M2 immunosuppressive states via Wnt signaling (57).
3. Checkpoint Dysregulation: Nuclear β-catenin/TCF4 complexes directly transcribe PD-L1 in intestinal-type GC with APC mutations, elevating PD-L1 expression 3.2-fold versus β-catenin-negative tumors (58, 59). This is exacerbated by EHD3 (a Wnt/EMT activator) which associates with poor prognosis and reduced CD8+ T-cell infiltration (58). Therapeutic synergy emerges from co-targeting: Disrupting Wnt signaling (e.g., β-catenin knockdown) enhances PD-1 antibody efficacy by increasing tumor cell apoptosis and CD8+ T-cell activity by 47% in co-culture models (59).

**Figure 2:**
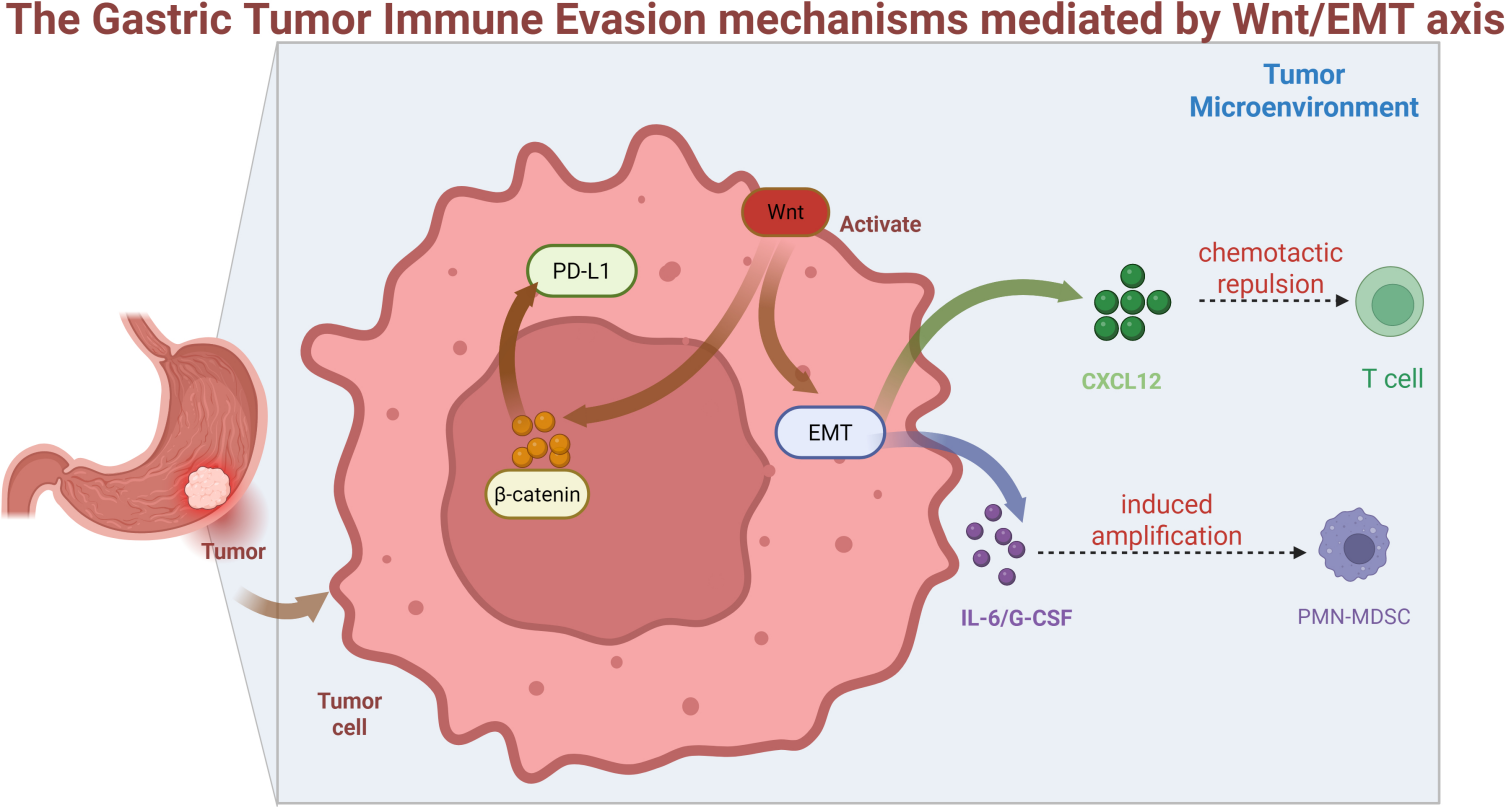
Wnt activation in tumor cells drives β-catenin nuclear translocation, inducing EMT and PD-L1 expression. EMT-derived CXCL12 secretion causes T-cell chemotactic repulsion, while IL-6/G-CSF release promotes PMN-MDSC expansion, collectively establishing an immunosuppressive tumor microenvironment.

**Figure 2**: These mechanisms establish Wnt/EMT-immune evasion as a therapeutic vulnerability, warranting combinatorial strategies discussed in Section 6.

## 6 Targeted therapy strategies and clinical potential

### 6.1 Natural compounds and synthetic drugs

Multiple phytochemicals demonstrate anti-gastric cancer activity through modulation of critical signaling pathways. Rutin, a bioactive flavonoid, exerts dose-dependent antitumor effects by suppressing Wnt/β-catenin signaling-mediated cellular proliferation, migration, and epithelial-to-mesenchymal transition (EMT) in gastric malignancies (60). The compound in question has been demonstrated to exhibit a dose-dependent antitumor effect. Dihydroartemisinin (DHA) has also been shown to impede the Wnt pathway, thereby hindering tumor progression. Additionally, honokiol has been shown to target *HDAC3*, inhibit epithelial plasticity (EP), and induce endoplasmic reticulum stress (61). Dihydroartemisinin (DHA) has also been shown to impede the Wnt pathway, thereby hindering tumor progression. Furthermore, honokiol has been observed to target *HDAC3*, inhibit epithelial plasticity (EP), and induce endoplasmic reticulum stress. In addition, gallic acid (GA) has been demonstrated to mitigate gastric precancerous lesions (GPL) by inhibiting Wnt/β-catenin signaling, thereby reversing EMT markers such as E-cadherin upregulation and N-cadherin/vimentin downregulation. Moreover, it has been demonstrated to block the transition from inflammation to cancer in both cellular and murine models (43, 62) Bidens pilosa-derived polyacetylene analogues (compounds 1-2) exhibit dual-pathway inhibition targeting both Wnt/β-catenin and Hippo/YAP axes, effectively suppressing metastasis through EMT marker modulation - specifically reducing vimentin/*snail* expression while enhancing E-cadherin levels (63). It has been demonstrated that the addition of berberine (BBR) to a patient’s treatment regimen has the capability of reversing the phenotypic transition of gastric cancer (GC). This outcome is attributed to the drug’s capacity to target the TGF-β/Smad, PI3K/Akt, and Wnt/β-catenin signaling pathways. This alkaloid restores epithelial characteristics through upregulation of E-cadherin/ZO-1 coupled with downregulation of mesenchymal markers including N-cadherin and TGF-β1 (64).

### 6.2 Gene and RNA-targeted therapy

Emerging genetic interventions demonstrate significant potential in gastric cancer (GC) management through modulation of Wnt/β-catenin signaling and associated pathways: Circ_0003789: This oncogenic circular RNA drives GC progression and EMT via Wnt/β-catenin activation. Its suppression markedly inhibits tumor growth and metastasis in preclinical models (65). lncRNA PCAT6/miR-15a Axis: PCAT6 functions as a competitive endogenous RNA (ceRNA) to promote proliferation through Wnt/β-catenin and RB/E2F pathway activation. Targeted PCAT6 inhibition reverses these oncogenic effects (66). The study’s findings suggest that the inhibition of metastasis and proliferation of gastric cancer (GC) is achieved by suppressing Wnt/β-catenin signaling through the downregulation of WISP2. This observation provides further evidence that this pathway could serve as a viable therapeutic target (67). The TTY15/let-7a-5p/Wnt1 axis has emerged as a pivotal regulatory factor in this process. onco-protective long non-coding RNA TTTY15, which is silenced by CRISPR/Cas9, has been demonstrated to disrupt the regulatory loop between let-7a-5p and Wnt1. This disruption, in turn, has been shown to attenuate Wnt/β-catenin-driven PTD and progression (68). *TET1*/*FOXO4* Axis: CRISPR-induced *TET1* overexpression sequesters β-catenin in the cytoplasm, inhibiting Wnt signaling while stabilizing *FOXO4* to regulate cancer stem cell (CSC) properties and EMT processes (69). *NCAPG* Targeting: *NCAPG* knockdown reverses Wnt/β-catenin activation, reduces EMT markers (e.g., *Snail* suppression with E-cadherin upregulation), and induces apoptosis in gastric adenocarcinoma (70). *FNDC1* Silencing: CRISPR-mediated *FNDC1* suppression inhibits Wnt/β-catenin signaling and EMT, effectively blocking peritoneal metastasis while offering dual diagnostic-therapeutic utility (71). The following investigation will address the *CXXC5*/*KANK1* regulation. The restoration of *CXXC5* or *KANK1* expression through CRISPR/Cas9 technology has been demonstrated to suppress Wnt/β-catenin/Axin2 signaling, reverse EMT, and promote apoptosis in germ cells (GCs). It has been demonstrated that synergistic effects emerge when combinatorial targeting strategies are employed (72). CRISPR/Cas9 has been demonstrated to facilitate the precise modulation of Wnt/β-catenin regulators, including *TET1* and NCAPG. This modulation has been shown to suppress ETP and metastasis (69, 70). The utilization of CRISPR/Cas9 technology in conjunction with non-coding RNAs (TTTY15) or epigenetic modifiers (*TET1*) results in the modification of oncogenic signaling networks, thereby enhancing the specificity of therapeutic interventions (68, 69).

### 6.3 Prognostic markers

Exosomal *circ0000670* and plasma *LINC01225*: Potential non-invasive diagnostic markers (23, 30, 33): The utilization of CRISPR/Cas9 technology in conjunction with non-coding RNAs (TTTY15) or epigenetic modifiers (*TET1*) results in the modification of oncogenic signaling networks, thereby enhancing the specificity of therapeutic interventions (61, 73). Elevated levels of *APOD* in gastric cancer (GC) have been shown to promote proliferation and metastasis via the Wnt/β-catenin/EMT axis. In addition, *APOD* has been linked to unfavorable prognoses, functioning as a prognostic biomarker associated with the remodeling of the tumor microenvironment (74). *TET1*/*FOXO4*: Reduced expression levels of *TET1* and *FOXO4* have been identified as a prognostic indicator for colorectal cancer, indicating a negative correlation with patient outcomes. The repression of these genes has been demonstrated to activate Wnt/β-catenin signaling, thereby enhancing the characteristics of the disease (69). The present study investigates the role of the long non-coding RNA (lncRNA) SNHG11 in colorectal cancer (CRC). The elevated expression of SNHG11 in CRC is associated with adverse outcomes by fostering EMT and autophagy through the modulation of microRNA (miRNA) correspondence, as also demonstrated by the activation of Wnt/β-catenin. This finding suggests that SNHG11 has the potential to serve as a therapeutic and prognostic indicator (75). Fosl1: The elevated expression of Fosl1 in colorectal cancer (CRC) and colon cancer has been demonstrated to be associated with a poor prognosis. This association is attributed to the promotion of metastasis that occurs through Smurf1-mediated activation of the Wnt/β-catenin signaling pathway and induction of epithelial-mesenchymal transition (EMT) (76). Asymmetric dimethylarginine (ADMA) is a serum marker that has been the focus of research in the context of gastric cancer. Elevated levels of ADMA have been demonstrated to be concomitant with diminished mortality rates in patients diagnosed with this condition. The role of ADMA in enhancing epithelial-to-mesenchymal transition (EMT) and metastasis through Wnt/β-catenin signaling pathways has been attributed to this phenomenon. It has been determined that this attribute establishes ADMA as a non-invasive prognostic biomarker (65). *SERPINH1*: The demonstration that *SERPINH1* expression in gastric cancer is associated with lymph node metastasis and poor survival outcomes has been well-documented. It is hypothesized that the association is mediated through the activation of the Wnt/β-catenin signaling and epithelial-mesenchymal transition (EMT) pathways (77), which has been established as a validated therapeutic and prognostic target (78). *LDLRAD2* upregulation in gastric cancer has been shown to promote metastasis through the Axin1/β-catenin axis-mediated Wnt activation and epithelial-mesenchymal transition (EMT). This upregulation functions as an independent prognostic indicator for advanced disease (79). These markers have been observed to interact with Wnt/β-catenin and epithelial-mesenchymal transition (EMT) signaling pathways, thereby facilitating metastasis. The dysregulation of these genes, whether through the process of over-expression or under-expression, has been demonstrated to be a significant factor in the progression of tumor stages to more advanced stages, the development of therapeutic resistance, and the subsequent reduction in survival outcomes (69, 74, 76, 79). It has been posited that the targeting of specific markers, including SNHG11 antisense oligonucleotides and ADMA inhibitors. The aforementioned findings have the potential to result in a reversal of the Wnt/β-catenin-mediated pathway associated with PTD (75, 77).

## 7 Challenges and future perspectives

### 7.1 Pathway complexity

The Wnt/β-catenin signaling pathway exhibits substantial cross-talk with Hedgehog, Notch, and TGF-β pathways, thereby establishing a dynamic network that contributes to tumor heterogeneity and therapy resistance in gastric cancer (GC) (80, 81). The complexity of therapeutic targeting is further compounded by context-dependent interactions between the Wnt pathway and other signaling pathways. These additional pathways include, but are not limited to, PI3K/AKT and Hippo/YAP. Inhibition of one pathway may result in compensatory activation of alternative oncogenic signals (7, 82). The complex interaction among the Wnt signal pathway and other signal pathways (e.g., PI3K/AKT and Hippo/YAP) presents substantial difficulties in the development of effective therapeutic interventions. It is imperative to note that the inhibition of these pathways has the potential to result in unintended consequences (8).

### 7.2 Delay in clinical translation

Despite the initial optimism fueled by the preliminary clinical data, the translation of Wnt-targeted therapies, such as PORCN inhibitors and β-catenin degraders, into clinical practice has proven to be fraught with substantial challenges. These challenges stem from issues related to off-target toxicity, inadequate bioavailability, and the absence of predictive biomarkers (83, 84). A mere 5% of Wnt-related gastric cancer studies progress to clinical trials, underscoring the necessity for patient-derived organoid models and three-dimensional bioprinting systems. Addressing the discrepancy between preclinical and clinical data is imperative (85, 86). The following essay will address the challenges associated with drug delivery. In the context of future research studies, the utilization of nanoparticle-based Wnt inhibitors and proteolysis-targeted chimeras (PROTACs) holds promise for enhancing specificity and reducing systemic toxicity (87).

The representative agents targeting Wnt/β-catenin in GC, spanning clinical and preclinical stages, are summarized in **Table 2**.

**Table 2:** Key Wnt/β-catenin-targeting agents in gastric cancer.

Agent	Type	Mechanism	Stage	Efficacy in GC
LGK974	Synthetic drug	Inhibits Wnt secretion	Clinical I/II	24% ORR in APC-mutant GC
Ginsenoside Rg3	Natural compound	Suppresses β-catenin nuclear translocation	Preclinical	Cisplatin IC50 reduced by 60%
IGC-001	Wnt inhibitor	Disrupts β-catenin/TCF4 complex	Preclinical	Restores trastuzumab sensitivity
5-Azacytidine	Epigenetic agent	Demethylates WIF1/Wnt7a	Preclinical	Suppresses lymph node metastasis

### 7.3 Individualized treatment

The UPS (ubiquitin-proteasome system) scoring system is a method of evaluating and stratifying gastric cancer patients according to the activity of the canonical Wnt/β-catenin pathway. The system under discussion has been demonstrated to facilitate the formulation of personalized therapy options, such as LGK974, for tumors that exhibit high levels of UPS activity (55). Combination strategies: The combination of Wnt/β-catenin targeting and immune Checkpoint Inhibitor (ICI) therapy (e.g., anti-PD-1) or Epigenetic Agent (EA) therapy (e.g., HDAC inhibitors) has demonstrated synergistic efficacy in preclinical models. This finding supports the commencement of phase II clinical trials (59, 88) Liquid biopsy-based monitoring: The analysis of circulating tumor DNA (ctDNA) for mutations in the Wnt pathway, including *CTNNB1* and *APC*, enables real-time modifications to therapeutic regimens, thereby addressing resistance (89). Clinical trials enriched with priority biomarkers (e.g., Fosl1 increased, *TET1* decreased) aim to evaluate the efficacy of Wnt-targeted therapy in molecularly defined subtypes of gastric cancer (90).

## 8 Discussion

In this review, a thorough investigation of the molecular mechanisms underlying the Wnt/β-catenin signaling pathway and epithelial-mesenchymal transition (EMT) in gastric cancer (GC) is presented, along with a discussion of the possible implications for therapy. In addition, we examine the essential roles of genetic, epigenetic, and non-coding RNA (ncRNA) regulatory networks in these processes. The ensuing analysis is structured into three primary domains: research advancements, obstacles in clinical application, and potential avenues for future investigation.

### 8.1 Discovery of core mechanism

An exhaustive investigation was undertaken to elucidate the molecular mechanisms through which genetic regulators, including *NAT10* and *SMC4*, modulate the Wnt/β-catenin signaling pathway via post-translational modifications (e.g., acetylation) or nuclear transportation processes. For instance, *NAT10* has been demonstrated to be significantly correlated with cisplatin resistance, a phenomenon that can be attributed, at least in part, to its capacity to stabilize β-catenin and to initiate a process known as epithelial-mesenchymal transition (EMT) (18), It has been demonstrated that the strategic targeting of *NAT10* could represent a promising approach to enhance chemotherapeutic sensitivity. Furthermore, non-coding RNAs (e.g., *LINC00665* and *circ0000670*) contribute significantly to tumor progression by interacting with critical molecules within the Wnt pathway (such as *Wnt3a* and HMGB1). It is imperative to note that exosomal particles, induced by the presence of cigarette smoke, have been observed to promote the development of precancerous lesions by stimulating the Wnt signaling pathway (33), This study provides novel insights into the regulatory mechanisms of tumorigenesis by environmental carcinogens through epigenetic pathways.

The findings of the present study offer a more exhaustive explanation of the mechanism through which epigenetic deregulation, for instance the hypermethylation of the *miR-33b* promoter, considerably exacerbates the epithelial-mesenchymal transition (EMT) and chemoresistance. This process involves interactions among multiple pathways, including NF-κB and Notch (44). The restoration of microRNA-33b expression has been identified as a potential strategy for impeding the Wnt/β-catenin signaling pathway, thereby effectively disrupting signals associated with metastasis promotion. The findings of the present study underscore the substantial potential of this receptor as a therapeutic target for combination treatments.

### 8.2 Exploration and limitations of therapeutic strategies

A thorough review of the existing literature was conducted to investigate the various approaches designed to target the Wnt/β-catenin-EMT pathway. The present analysis encompassed a comprehensive range of substances, including, but not limited to, natural substances such as rutin and ginsenoside Rg3. Furthermore, the review also incorporated state-of-the-art genetic engineering methods, including CRISPR/Cas9 targeting *TET1/FOXO4*. For instance, ginsenoside Rg3 was found to decrease the IC50 value of cisplatin by 60% through the suppression of β-catenin nuclear localization (46). Concurrently, the knockout of the *FNDC1* gene exerted a substantial inhibitory effect on peritoneal metastasis (71). Nonetheless, the implementation of these strategies in clinical practice remains challenging due to issues such as off-target effects associated with CRISPR technology and inadequate bioavailability of natural compounds, which underscore the necessity for further refinement (83, 84).

The integrated treatment regimens that were proposed, including the concurrent administration of the Wnt inhibitor ICG-001 and PD-1 inhibitors, have demonstrated a synergistic effect in preclinical studies (59). The effectiveness of this method in patients with gastric cancer, however, remains to be validated through further studies. Moreover, the observation of alterations within the Wnt signaling pathway (e.g., *CTNNB1* and *APC*) through liquid biopsy possesses the capability to enable the real-time adjustment of therapeutic regimens (89).

### 8.3 Challenges and future directions

It has been established that there exists a complex relationship between the Wnt pathway and an array of other signaling networks, including the PI3K/AKT and Hippo/YAP networks (7, 82). It is conceivable that this may result in compensatory activation subsequent to the inhibition of a singular pathway. For instance, the suppression of Wnt signaling may lead to the compensatory activation of the Hedgehog or Notch pathways (80, 81). It is conceivable that this may result in compensatory activation subsequent to the inhibition of a singular pathway. For instance, the suppression of Wnt signaling may result in the compensatory activation of the Hedgehog or Notch pathways.

With regard to biomarkers, exosomal *circ0000670* and plasma *LINC01225* have been shown to possess promising diagnostic potential (30, 33), their prognostic relevance in subtypes of gastric cancer (GC) requires validation through large-scale cohort studies. We propose combining multi-omics data, including scores from the ubiquitin-proteasome system (55), with patient-derived organoid models (85, 86) to promote accurate classification and the creation of personalized treatment approaches.

## 9 Conclusion

Recent studies have identified the Wnt/β-catenin signaling pathway as a critical factor in the development of gastric cancer, as well as in the promotion of tumors and chemoresistance. The targeting of key molecules within this pathway—such as *NAT10*, circadian proteins, and *HDAC3*—or the employment of combinations of natural products may provide solutions to the current therapeutic challenges. In order to move forward, it is essential to enhance mechanistic research, foster interdisciplinary strategies, and facilitate the clinical translation of precision treatments for gastric cancer.
